# Differentiation of Induced Pluripotent Stem Cells into Male Germ Cells *In Vitro* through Embryoid Body Formation and Retinoic Acid or Testosterone Induction

**DOI:** 10.1155/2013/608728

**Published:** 2012-12-30

**Authors:** Peng Li, Hongliang Hu, Shi Yang, Ruhui Tian, Zhenzhen Zhang, Wei Zhang, Meng Ma, Yong Zhu, Xizhi Guo, Yiran Huang, Zuping He, Zheng Li

**Affiliations:** ^1^Shanghai Human Sperm Bank, Department of Urology, Shanghai Institute of Andrology, Renji Hospital, Shanghai Jiao Tong University School of Medicine, Shanghai 200001, China; ^2^Clinical Stem Cell Research Center, Renji Hospital, Shanghai Jiao Tong University School of Medicine, Shanghai 200127, China; ^3^BIO-X Center, Shanghai Jiao Tong University, Shanghai 200240, China; ^4^Shanghai Key Laboratory of Reproductive Medicine, Shanghai Jiao Tong University School of Medicine, Shanghai 200025, China; ^5^State Key Laboratory of Oncogenes and Related Genes, Shanghai Cancer Institute, Renji Hospital, Shanghai Jiao Tong University School of Medicine, Shanghai 200032, China

## Abstract

Generation of germ cells from pluripotent stem cells *in vitro* could have great application for treating infertility and provides an excellent model for uncovering molecular mechanisms controlling gametogenesis. In this study, we explored the differentiation potential of mouse induced pluripotent stem (iPS) cells towards male germ cells. Embryoid body formation and retinoic acid/testosterone induction were applied to promote differentiation of mouse iPS cells into male germ cells *in vitro*. Quantitative RT-PCR and immunoflourescence were performed to characterize the iPS cell differentiation process, and notably there were different temporal expression profiles of male germ cell-associated genes. The expression of proteins, including MVH, CDH1, and SCP3, was remarkably increased. mRNA expression of *Stra8*, *Odf2*, *Act*, and *Prm1* was upregulated in iPS cells by retinoic acid or testosterone induction, whereas *Oct-4* transcription was reduced in these cells compared to the controls. Hormones were also measured in the EB medium. DNA content analysis by flow cytometry revealed that iPS cells could differentiate into haploid cells through retinoic acid or testosterone treatment. Collectively, our results suggest that mouse iPS cells possess the potency to differentiate into male germ cells *in vitro* through embryoid body formation and retinoic acid or testosterone induction.

## 1. Introduction

Male germ cells play a critical role in transmitting genetic information to the offspring by combining with the female germ cells through the unique process of fertilization. Gametogenesis is a process in which a diploid precursor becomes a haploid germ cell. Any error at any stage of the gametogenesis process results in subfertility or infertility, which is a major public health issue affecting about 10–15% of couples [1]. As an example, azoospermia is observed in 1% of the general population and in 10–15% of infertile men [2, 3]. Furthermore, nonobstructive azoospermia, resulting from a testicular failure, affects about 10% of infertile men and is diagnosed in 60% of azoospermic men [2, 4]. However, little is known about molecular mechanisms underlying gametogenesis due to the lack of an efficient and reproducible model representing gametogenesis.

Recently, considerable progress has been made in the derivation of germ cell from embryonic stem cells (ESCs), which are regarded as a desirable experimental model for elucidating mammalian germ cell development and potential strategies for producing haploid germ cell. In mice, Hübner et al. first reported the successful derivation of gametes from mouse ESCs *in vitro* [5]. This is a significant breakthrough, and it has important impact on the study of germ cell development. Furthermore, Nayernia et al. showed the first live offspring of mice from intracytoplasmic sperm injection (ICSI) using sperm induced from ESCs *in vitro* [6]. Mouse ESCs can be induced to generate motile and tailed sperm by ectopic expression of Dazl [7]. In human, differentiation of germ cell from human ESCs has also been demonstrated [8–14]. Undoubtedly, ESCs possess the capacity to differentiate into sperm. Generally, there are two methods to produce sperm from the ESCs *in vitro*, namely, the monolayer differentiation and the embryoid body (EB) formation [15]. EB is a three-dimensional cellular aggregate including a mixture of cells from the endoderm, the ectoderm, and the mesoderm when the ESCs are cultured in a condition without any differentiation inhibitors.

However, there are ethical problems in obtaining ESCs which results in a limited resource of ESCs. One of the exciting breakthroughs in stem cell biology is establishment of the induced pluripotent stem (iPS) cells from somatic cells by transferring pluripotent genes, including *Oct4*, *Sox2*, *c-Myc*, and *Klf4* [16]. Notably, iPS cells have advantages over ESCs in the following aspects: (1) there is no ethical issue using human somatic cells; (2) the source of human somatic cell is abundant. The iPS cells can give rise to all types of cells including the germ cells [16]. Significantly, iPS cells were able to generate viable, live-born offspring through tetraploid complementation [17], demonstrating that iPS cells also have a similar developmental pluripotency with ESCs. Mouse iPS cells could differentiate into hematopoietic precursor cells that have been used to rescue the mice with sickle cell diseases [18]. Recent studies have demonstrated the feasibility of *in vitro* differentiation systems for germ cell derivation from iPS cells. The iPS cells derived from mouse adult hepatocytes were able to be induced into primordial germ cells [19]. Park et al. also reported that human iPS cells could differentiate into primordial germ cells when cocultured with human fetal gonadal cells [20], and mouse iPS cells could differentiate into epiblast-like cells that further generate primordial germ cell-like cells by treatment with BMP4 [21]. However, it is not yet known whether iPS cells derived from fibroblast cells could spontaneously produce male germ cells or with retinoic acid (RA) treatment. Fibroblast cells could be obtained easily, and patient-derived iPS cells could be used for patient-specific therapy without immune rejection. Therefore, we explored the capability of fibroblast-derived iPS differentiation into male germ cell *in vitro* using embryoid body formation and retinoic acid/testosterone induction. Our data suggest that iPS cells can differentiate into spermatogonial stem cells and late stages of male germ cells, which could provide an ideal platform to uncover the mechanisms regulating spermatogenesis and open novel possibilities for using male germ cells derived from patient-derived iPS cells in treating male infertility in future. 

## 2. Materials and Methods

### 2.1. Mouse iPS Cells and Culture

Mouse iPS cell line (Tg-GFP-miPS11.1; 40, XY; [22]) was a kind gift from Professor Ying Jin (Shanghai Jiao Tong University School of Medicine). These iPS cells originated from MEF cells by retroviral transduction of *Oct4*, *Sox2*, *c-Myc*, and *Klf4*. Mouse iPS cells were cultured with the high-glucose DMEM supplemented with 15% FBS (Thermo Scientific HyClone, South Logan, UT, USA), 0.1 mM nonessential amino acids, 2 mM L-glutamine, 0.1 mM 2-mercaptoethanol, 100 U/mL penicillin, 100 mg/mL streptomycin (Invitrogen, San Diego, CA, USA), and LIF (Millipore, Billerica, MA, USA), on feeder cells in gelatinized dishes. The cells were passaged every 2-3 days from passages 16 to 30.

### 2.2. Spontaneous Differentiation of iPS Cells and Retinoic Acid or Testosterone Induction

After being digested with trypsin-EDTA, ~1 × 10^6^ iPS cells were transferred to 10 cm Petri dishes containing 10 mL of the above culture medium without LIF. EBs were formed using the handing drop method and maintained in suspension. At day 3, half of the medium was changed every 2 days. In addition, 2 *μ*M retinoic acid (RA, Sigma, St. Louis, MO, USA) or 1 *μ*M testosterone (Sigma) or 2 *μ*M RA and 1 *μ*M testosterone combination was added into culture medium at day 5. Forty-eight hours later, the medium was changed, and the cells were cultured for two weeks.

### 2.3. RNA Isolation, cDNA Synthesis, and Quantitative PCR

The iPS cells and EBs (at days 0, 4, 7, and 14) were digested with trypsin and collected by centrifugation. Total RNA was extracted using Trizol (Invitrogen, San Diego, CA, USA) according to the manufacturer's protocol. cDNA was generated from 2 *μ*g of total RNA using random hexamer primers under standard conditions (Promega Biotech Co., San Luis Obispo, CA, USA). For quantitative PCR (qPCR), SYBR Green master mix (Life Technologies Corporation, Carlsbad, CA, USA) was added to each well of the PCR plate (10 *μ*L of SYBR Green, 6 *μ*L of water, 2 *μ*L of primers, and 2 *μ*L of cDNA), according to the following procedure: 40 cycles at 95°C for 30 s, 60°C for 45 s, and 72°C for 60 s. Samples were run in triplicates. qPCR data and relative quantification were analyzed by Graphpad Prism5 for windows. According to the delta-Ct method, the threshold of cycle values was normalized against the threshold value of mouse housekeeping gene *Gapdh*. The list of gene primer for the selected genes was from the [23] and shown in Table 1. 

### 2.4. Western Blots

Total proteins were extracted from EBs using the RIPA lysis buffer supplemented with a mixture of PMSF, sodium orthovanadate, and protease inhibitors (Promega Biotech Co.). After 30 min lysis on ice, cell lysates were cleared by centrifugation at 12,000 rpm, and the concentration of protein was measured by the Bio-Rad Bradford assay with BCA as the standard. For Western blots, 30 *μ*g of cell lysate from each sample was used for SDS-PAGE and transferred to nitrocellulose membranes. The membrane was probed with rabbit polyclonal to the MVH antibody (Catalog : ab13840, 1 : 100 dilutions, Abcam, Cambridge, CB, UN), and goat anti-mouse secondary antibodies were used. The antibody-antigen complexes in the membranes were visualized using an enhanced-chemiluminescent detection kit (Santa Cruz Biotechnology Inc., Santa Cruz, CA, USA).

### 2.5. Immunofluorescence Staining

EBs derived from iPS cells were fixed in 4% paraformaldehyde in PBS and permeabilized with 0.4% Triton X-100 in PBS. After blocking, the primary antibodies against MVH (abcam), CDH1 (LifeSpan Biosciences Inc., Seattle, WA, USA), and SCP3 (abcam) were diluted at 1 : 100 and incubated for 2 hours at room temperature before staining with PE-conjugated secondary antibodies. Slides were examined under confocal fluorescence microscopy (Leica Microsystems CMS GmbH, Mannheim, Germany).

### 2.6. Measurement of Estradiol, Testosterone, and Gonadotropin from EB Medium

Three batches of EBs were cultured with EB medium. Half of medium was changed every 2 days. About 3 mL of the medium was collected after 4, 7, and 10 days of culture and stored at −20°C. The fresh EB medium was used as the control. The concentrations of testosterone, estradiol, and gonadotropin were determined by radioimmunoassay (RIA, courtesy of Dr. Kejia Gao, Department of Nuclear medicine, Central Hospital of Huangpu District, Shanghai, China).

### 2.7. Fluorescence-Activated Cell Sorting (FACS) Analysis

The iPS cell-derived EBs were digested by 0.25% trypsin-EDTA (Gibco) to obtain single iPS cell suspension. Cells were incubated with PE anti-mouse SSEA1 (Biolegend, San Diego, CA, USA) for 1 hour at room temperature. The cells were washed twice with PBS and analyzed with an FACS Calibur system (BD, Franklin Lakes, NJ, USA). The cells without primary antibody but with mouse IgG conjugated to PE were used as controls.

### 2.8. DNA Content Analysis by Flow Cytometry

The cells derived from iPS cells were collected and fixed with 70% ethanol for 1 hour at room temperature. These cells were incubated with a staining solution 0.1% Triton X-100, 0.2 mg/mL of RNase A and 0.02 mg/mL of propidium iodide (Invitrogen) for 15 min at 37°C. The cell was resuspended, and the DNA profile was analyzed on a Becton-Dickinson FACS Calibur (BD) according to procedure as described previously [8].

### 2.9. Statistical Analysis

All values were presented as mean ± SEM from three independent experiments, and statistically significant differences (*P* < 0.05) were determined among various groups by ANOVA and Tukey posttest using SPSS 12.0 statistical software.

## 3. Results

### 3.1. Expression of Germ Cell-Associated Genes during Spontaneous Differentiation of iPS Cells into EBs

**Figure 1:**
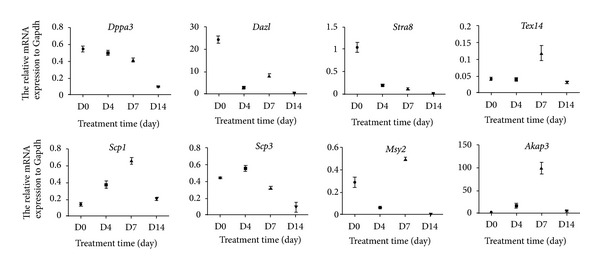
The expression profiles of germ cell-associated genes during EB formation from iPS cells. Real-time PCR was performed using cDNA from EB cultures maintained for 0, 4, 7, and 14 days (D0, D4, D7, and D14) to detect the relative mRNA expression of *Dppa3*,* Stra8*,* Tex14*,* Dazl*,* Scp1*,* Scp3*,* Msy2*, and *Akap3*. The mean normalized expression of each gene relative to that of *Gapdh* was shown.

A total of 8 genes were analyzed during EB formation. Among them, *Dppa3* (also called *Stella*) is a marker for cell pluripotency, and *Dazl *(deleted in azoospermia-like) and *Tex14* (testis expressed 14) represent the state of premeiotic stage of the cells [23]. *Scp1* (synaptonemal complex protein 1) and *Scp3* (synaptonemal complex protein 3) are markers of meiosis [15], while *Msy2* (also called Ybx2, Y box protein 2) and *Akap3* (A kinase anchor protein 3) are only expressed in the haploid germ cells [23]. *Stra8* (stimulated by retinoic acid gene) represents the ability of the cells' responding to retinoic acid which is accumulated in the premeiotic germ cells [15]. The expression patterns of these 8 genes were summarized in Figure 1. We found that, during EB formation, the expression of *Dppa3* and* Stra8* was decreased dramatically. In contrast, expression of *Scp1*, *Scp3* and transcripts of *Akap3* and *Msy2* were enhanced from day 4 to day 7 of EB formation. These results suggest that mouse iPS cells possess the potency differentiated into haploid male germ cells *in vitro *at day 7 after EB formation phenotypically.

### 3.2. Expression of the Germ Cell-Associated Proteins during Spontaneous Differentiation of iPS Cells into EBs

**Figure 2:**
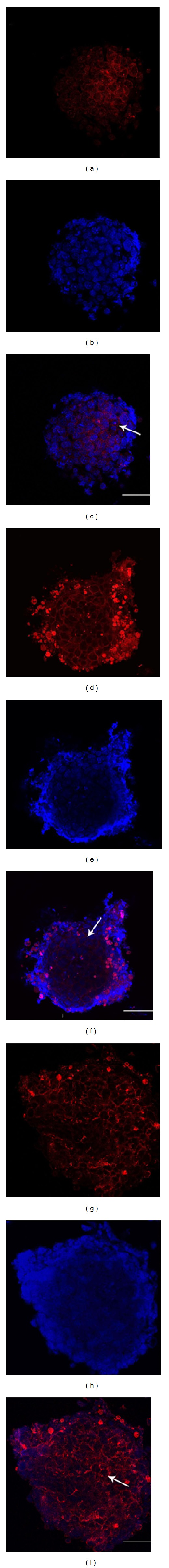
Expression of male germ cell markers in EBs derived from iPS cells. Expression of MVH (a), CDH1 (d), and SCP3 (g) in the EBs after cultured for 4–7 days was detected by immunofluorescent staining. DAPI (b, e, h) was used to indicate cell nuclei, and merged pictures (c, f, i) were shown. Arrows indicated the cells that were positive for MVH, CDH1, or SCP3. Scale bars = 50 *μ*m.

**Figure 3:**
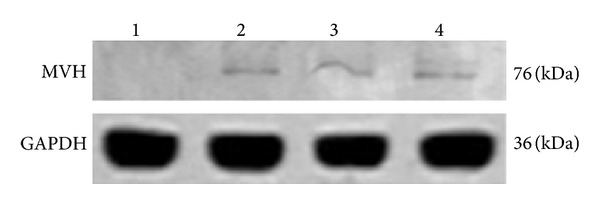
Western blot analysis of MVH (VASA) expression in iPS cells and EBs derived from iPS cells. Protein lysates from iPS cells (1), 4-day EBs (2), 7-day EBs (3) and 10-day EBs (4) were blotted and stained by MVH antibody.

The expression of male germ cell-associated proteins (MVH, CDH1, and SCP3) was also analyzed during EB formation from iPS cells by immunofluorescence. MVH, also namely VASA, is encoded by *Ddx4* for a DEAD box polypeptide 4, and it is a marker for male germ cells. CDH1 is encoded by Cdh1 for e-cadherin, and it has been regarded as a maker for spermatogonial stem cells in mice. SCP3 is the product of* Scp3* which is synaptonemal complex protein 3. As shown in Figure 2, we revealed that the expression of male germ cell marker MVH, spermatogonial stem cell marker CDH1, and synapsis marker SCP3 was expressed in the EBs after cultured for 4–7 days. These data indicate that the iPS cells we used possess the potential to differentiate into male germ cells, spermatogonial stem cells, and spermatocytes. We further detected MVH expression of iPS cells and EBs at day 4, day 7, and day 10 by Western blots. As shown in Figure 3, we found that expression of MVH started at day 4 of EB formation and was maintained for 10 days. 

### 3.3. Measurement of Estradiol, Testosterone, and Gonadotropin from EB Medium

We determined the concentrations of estradiol, testosterone, and gonadotropin of EB medium using radioimmunoassay. We found that level of estradiol was increased to 317 ± 57 pmol/mL in EB culture for 10 days (50% replacement of medium every 2 days; 7.5 mL medium per flask), while the estradiol was not detected in control medium. Neither testosterone nor chorionic gonadotropin was detected in culture or control medium. These results suggest that estradiol plays a role in the differentiation of PS cells into male germ cells. 

### 3.4. Effect of RA/Testosterone on iPS Cell Differentiation towards Male Germ Cells

**Figure 4:**
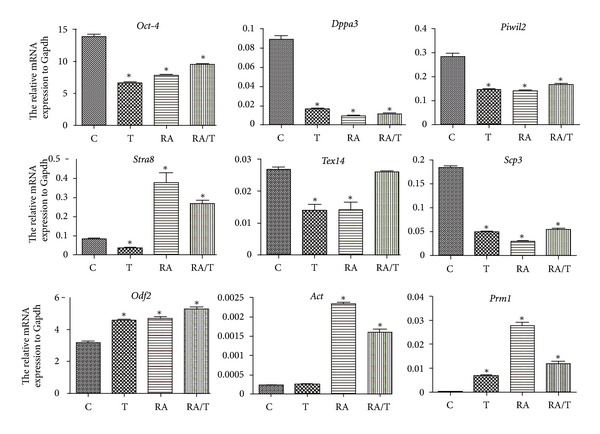
The expression profiles of germ cell-associated genes in iPS cells treated with RA or testosterone *in vitro*. qPCR was carried out using the cDNA of iPS cells in the presence or absence of RA and testosterone. The mean normalized expression of each gene relative to that of *Gapdh* was shown along the *y*-axis. C: the control group without retinoic acid or testosterone induction. T: the testosterone induction group. RA: the retinoic acid induction group. RA/T: the retinoic acid and testosterone induction group. Note: *indicated statistically significant differences (*P* < 0.05) in the mRNA expression between the RA- or testosterone-treated groups and the control.

**Figure 5:**
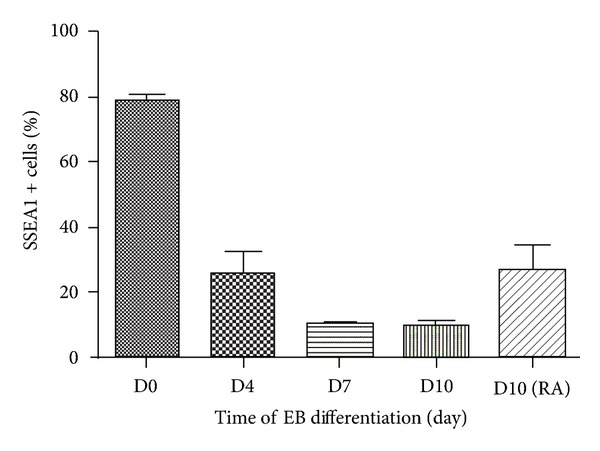
The percentage of SSEA1-positive cells during EB differentiation and RA induction. D0: the percentage of SSEA1-positive cells in iPS cell clones. D4: the percentage of SSEA1-positive cells in day 4 EBs. D7: the percentage of SSEA1-positive cells in day 7 EBs. D10: the percentage of SSEA1-positive cells in day 10 EBs. D10 (RA): the percentage of SSEA1-positive cells after RA induction.

**Figure 6:**
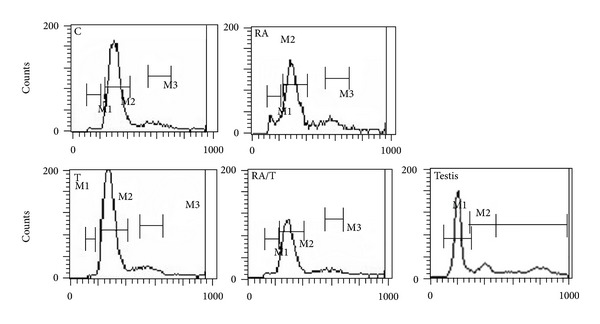
Flow cytometry showed DNA content in EBs derived from iPS cells with RA or testosterone *in vitro.* EBs were dispersed as single cells and stained with the DNA-binding dye Hoechst 33342 to reveal DNA content, and the cell debris was removed by setting the gates. M1, haploid cells; M2, diploid cells; M3, tetraploid cells. C: control group, M1 (1.15%), M2 (77.72%), and M3 (9.31%); RA: RA induction group, M1 (8.31%), M2 (61.69%), and M3 (12.98%); T: testosterone induction group, M1 (0.24%), M2 (81.27%), and M3 (10.17%); RA/T: RA and testosterone induction group, M1 (2.56%), M2 (74.49%), and M3 (8.86%); testis: testicular cells, M1 (59.67%), M2 (15.07%), and M3 (25.68%).

We further revealed that the expression of *Oct-4*, a marker for iPS cells, was significantly decreased by RA or testosterone induction (Figure 4). Conversely, the expression of *Stra8* was significantly increased by the addition of RA. Notably, the transcripts of haploid cell markers *Odf2*, *Act*, and *Prm1* [15] were enhanced significantly under RA or testosterone stimulation. There is a decrease for the expression of *Tex14* and *Scp3* in iPS cells in response to RA or testosterone alone. However, RA and testosterone combination induced an increase of *Tex14* expression, suggesting that RA and testosterone induce the differentiation of iPS cells into premeiotic male germ cells. SSEA is a marker for primordial germ cells, and interestingly we revealed that the percentage of SSEA1-positive cells was increased from 9% to 26% through RA treatment (Figure 5). Furthermore, flow cytometry showed that 2–8% of the cells were haploid cells after RA or testosterone induction (Figure 6). Collectively, these results indicate that iPS cells could differentiate into haploid male germ cells with RA or testosterone stimulation combined with EB formation.

## 4. Discussion

The iPS cells can be obtained through the introduction of defined factors into somatic cells. These cells are thought to resemble ESCs based on global gene expression analyses and possess the potential to differentiate into germ cells when placed in the proper environment *in vivo* [17, 24, 25]. However, few studies have tested the iPS cells' ability and efficiency in differentiation towards germ cells *in vitro*. Imamura et al. reported that iPS cells derived from mouse adult hepatocytes were able to be induced into primordial germ cells [19]. However, it is hard for the surgeons to obtain the hepatocytes of patients. Notably, fibroblasts are easy to obtain, and thus differentiation of iPS cells derived from fibroblasts into male germ cells is more convenient to patient-specific therapy. Hayashi et al. showed that mouse iPS could reconstitute the mouse germ cell specification pathway [21]. The iPS cell lines were generated by different somatic cells [26], and not all iPS cell clones can contribute to the germline [25, 27]. In the current study, two iPS cell lines (Tg-GFP-miPS11.1 and Tg-GFP-miPS4.1) were applied, and only the Tg-GFP-miPS11.1 iPS line showed the potential of differentiation into male germ cells. Thus, it is essential to select proper iPS cell line(s) with proven potential of contribution to germ cells. 

Previous studies have demonstrated the upregulation of late meiotic germ cell markers in ESCs by differentiation induction [7, 15, 23, 28]. Our q-PCR data demonstrated that there were downregulation of *Dppa3* and upregulation of premeiotic germ cell marker (*stra8*), meiotic germ cell markers (*Scp1*, *Scp3*, and *Msy2*), and haploid cell marker (*Akap3*, *Act*, *Odf2*, and *Prm1*) [15] when iPS cells are differentiated. During iPS cell spontaneous differentiation period, the expression profiles of *Scp1* and *Scp3* were not identical, and they reached peak at different time point. The expression profile of *Scp3* was consistent with Clark et al.'s findings [10], but Silva et al. reported that *Scp1* and *Scp3* had opposite expression profiles [23]. Furthermore, Lin et al. reported that *Dazl* has an obligatory function upstream of *Stra8* expression [29], and thus, there are similar expression patterns of *Stra8* with that of *Dazl* during EB formation. 

In this study, we revealed that both in the spontaneous and RA-induced differentiation, the iPS cells could enter different germ differentiation stages. We found that during 4–7 days of EB formation, some cells in the middle of EB expressed MVH (a germ cell marker), whereas iPS cell clones did not express MVH, which is similar to ESCs [30]. Some of the differentiated cells expressed CDH1, a specific spermatogonial stem cell protein [31], and other cells are positive for SCP3, a special marker for meiosis. Previous studies have shown that MVH is a specific marker for male germ cells from E10.5 to E17.5 [32] and from spermatogonia to the post-meiotic stage [19, 30]. MVH is considered as the most reliable germ cell-specific maker. Furthermore, Eguizabal et al. demonstrated that RA could promote the complete meiosis from human iPS cells, and the iPS-derived germ cells express MVH (VASA) [33]. Meiotic spreads were classified as punctate or elongated SCP3 staining patterns, corresponding to the early leptotene stage (punctate) and the later zygotene, pachytene, and diplotene stages (elongated) of meiotic prophase I [34]. In this study, most of cells were positive for SCP3, which indicates that these cells were the elongated male germ cells. This was verified by our q-PCR data that expression of *Tex14*, *Scp1*, *Akap3*, and *Msy2* reached peak at day 7 of EB formation. Qin et al. also proved that germ cell-associated markers were expressed in EBs from day 3 [35], and Toyooka et al. reported that about 0.4 ± 0.2% of the EB cells were MVH positive from day 5 to day 7 during spontaneous differentiation period [30]. All these data indicate that iPS cells could differentiate into male germ cells, and from day 4 to day 7 is the best period to add the inducer for iPS cell induction.

Significant efforts by numerous labs worldwide have established the roles for RA, SCF/kit, and BMPs to induce stem cells into germ cells [36]. RA can promote the progression of spermatocytes through early stages of meiosis and iPS cells towards primordial germ cells [20, 24, 25, 33], and thus, it was chosen as the inducing agent in this study. RA is a small and polar molecule that easily diffuses through tissues and acts by binding to nuclear RA receptors (RARs), which heterodimerize with nuclear retinoid X receptors (RXR) [37, 38]. RAR-RXR dimers bind to RA-response elements (RAREs) and thereby control the expression of RA-responsive genes [37]. It has been shown that exposure to RA controls whether mouse fetal germ cells enter meiosis or not [39, 40]. In our study, the final RA concentration is 2 *μ*M, which was consistent with Silva et al.'s study [23]. RA could be applied to select SSEA1-positive germ cells from the EBs [15]. In our study, the percentage of SSEA1-positive cells was increased from 9% to 26% after 48 hours of RA treatment.

Testosterone secreted by the Leydig cells is required for spermatogenesis *in vivo*, and it acts on Sertoli cells to stimulate gene transcription and produce growth factors that promote germ cell differentiation [41]. Testosterone was not detectable above background in our study, but this hormone can be aromatized to estradiol, which is crucial for spermatogenesis. Level of estradiol was increased in medium to 317 ± 57 pmol/mL in EB culture for 10 days. Silva et al. reported that a combination of RA and testosterone could induce mouse ESCs into germ cells [23], and thus, testosterone was applied to induce iPS cells into germ cells. Testosterone significantly decreases the expression of iPS cell marker *Oct-4*, whereas the expression of haploid cell markers *Odf2*, *Act*, and *Prm1* was increased significantly. Testosterone may act on Sertoli cells to produce growth factors including stem cell factor (SCF) that is required for differentiation of spermatogonial stem cells into male germ cells [41].

Since *Stra8* is the target gene of RA, the expression of *Stra8* is suppressed in absence of RA, and with RA addition, *Stra8* is successfully stimulated in the study. Furthermore, the pluripotent stem cell marker *Oct-4* is suppressed. However, the response of premeiotic and the postmeiotic markers with RA stimulation is different. The postmeiotic markers (*Odf2*, *Act*, and *Prm1*) [6, 15] are stimulated markedly. It is suggested that RA exerts effects via targeting *Stra8* to promote the haploid germ cell formation during EB formation. Recent study has demonstrated that the iPS cells could be induced into primordial germ cells. However, there is so far not a perfect stimulation protocol for iPS cells towards functional gametes *in vitro* available. In the current study, about 2–8% of the EB cells were haploid cells after RA or testosterone induction, while West et al. reported that about 12% of the cells were haploid cells [15]. 

In summary, we have demonstrated that mouse iPS cells could differentiate into male germ cells, even haploid cells through EB formation and RA or testosterone induction. The ability to generate male germ cells from human iPS cells derived from fibroblasts provides an excellent paradigm for elucidating the mechanism of gametogenesis as well as for developing new approaches for treating male infertility. 

## Figures and Tables

**Table 1:** Primers for q-PCR analysis of male germ cell markers.

Gene	Forward sequence (5′-3′)	Reverse sequence (5′-3′)
*Oct-4*	CCCTCTGTTCCCGTCACTG	ACCTCCCTTGCCTTGGCT
*Dppa3*	TGTCGGTGCTGAAAGACCCTAT	TTTCCTTCGAGCCTTTTTTGTC
*Piwil2*	TGACCTGTGCATCCCCTTCT	TCCCCACAAGCTTCATATCCA
*Tex14 *	GCGTATCGCAGTCGGCA	CCATGTGCAGCACTGGGA
*Stra8*	GTTTCCTGCGTGTTCCACAAG	CACCCGAGGCTCAAGCTTC
*Dazl*	AATGTTCAGTTCATGATGCTGCTC	TGTATGCTTCGGTCCACAGACT
*Scp1*	CGCTACAACCACATGCTTCG	GGAACGCTGCTTAGATCTCCTC
*Scp3*	ATGCTTCGAGGGTGTGGG	TTCCACCAGGCACCATCTTT
*Msy2*	CACCAAGGAGGATGTCTTTGTTC	CCAACACTCCGCAGAAACTTC
*Odf2*	CTGCCTTGTTAAGGTGTTGATGTC	TCATGGCCTTGAAGGATACCA
*ACT*	GTGTGCAGCCTGCACCAA	ACTGGCGGTCTTGAAAGCA
*Akap3*	ACGCCACTTTGACTTTGTAACCA	AAGACACCAATAAGGCTCATTCG
*Prm1*	AGGTGTAAAAAATACTAGATGCACAGAATAG	TTCAAGATGTGGCGAGATGCT
*Gapdh*	AGA ACATCATCCCTGCATCC	CACATTGGGGGTAGGAACAC
